# Bilinguals have better recall for code-switched information

**DOI:** 10.3758/s13423-025-02823-1

**Published:** 2026-02-11

**Authors:** Lauren K. Salig, Jorge R. Valdés Kroff, Jared M. Novick, L. Robert Slevc

**Affiliations:** 1 Program in Neuroscience and Cognitive Science, University of Maryland, College Park, MD USA; 2 Department of Psychology, University of Michigan, Ann Arbor, MI USA; 3 Department of Spanish and Portuguese Studies, University of Florida, Gainesville, FL USA; 4 Department of Hearing and Speech Sciences, University of Maryland, College Park, MD USA; 5 Department of Psychology, University of Maryland, College Park, MD USA

**Keywords:** Bilingualism, Code-switching, Comprehension, Memory

## Abstract

Bilinguals frequently code-switch during conversations—a behavior often viewed as creating processing challenges for listeners. However, code-switching may also enhance comprehension and memory by directing attention to key information. This study tested whether bilinguals recall information better in code-switched contexts compared with single-language contexts and explored whether code-switching experience amplifies this benefit. In a preregistered study, Spanish–English bilinguals listened to short vignettes containing both single-language and code-switched segments. Participants recalled details more accurately when they had been presented within a code-switched sentence, suggesting that switches act as cues that boost attention and memory encoding. Moreover, bilinguals with greater everyday code-switching experience showed the strongest recall benefits, supporting the idea that listeners learn to associate switches with communicative importance. These findings challenge the long-standing view that code-switching primarily imposes cognitive costs in comprehension. Instead, they reveal how bilinguals leverage the communicative value of code-switches to enhance memory for linguistic content. By demonstrating that code-switches can promote learning and retention, this study highlights the potential for code-switching to serve as a communicative tool, particularly in contexts where understanding and recalling information is critical.

## Introduction

In monolingual interactions, sentences begin and end in the same language. In bilingual interactions, however, a sentence can start in one language and finish *en otro idioma*. Psycholinguistic research has shown that processing these *code-switches* can be challenging for bilingual listeners. Compared with single-language sentences, code-switched sentences often elicit longer processing times and neural responses indicative of difficulty (Altarriba et al., 1996; Bultena et al., 2015; Moreno et al., 2002). These purported “switch costs” align with societal narratives that language mixing is confusing and best avoided, particularly in educational and parenting contexts (for critiques, see Baralt & Mahoney, 2020; Lin, 2013).

We challenge this deficit-focused narrative by revisiting how code-switching is studied. Traditional studies on the effects of code-switching on comprehension often measure performance, such as reading times, on individual trials, where each trial is treated as independent from the next. While this approach is valuable for isolating the costs of code-switches, it overlooks the broader communicative context in which code-switching naturally occurs—conversations and narratives where speakers use switches purposefully to convey meaning or emphasize key information, in addition to signaling bilingual identity and in-group membership. By focusing on single-trial paradigms, these studies may miss how bilinguals integrate code-switches over time to support comprehension and memory. In contrast, our approach shifts towards studying code-switching in more naturalistic contexts, where switches function not as isolated disruptions but as integral elements of effective communication.

Although switch costs are well-documented, research increasingly shows that these costs diminish or even disappear in more naturalistic settings (e.g., Kaan et al., 2020; Salig et al., 2024). Moreover, code-switching may offer comprehension benefits. For instance, code-switches often occur with lower frequency and with new or unexpected information (Calvillo et al., 2020; Myslín & Levy, 2015), allowing bilinguals to use switches as discourse cues to predict upcoming content (Tomić & Valdés Kroff, 2022). Switches can also enhance attention and memory. Bilingual listeners recognize information more accurately if they heard it earlier within a code-switched narrative (Salig et al., 2025), and young bilingual children show improved vocabulary learning in code-switched contexts (Blair & Morini, 2023; Read et al., 2021; cf. Byers-Heinlein, 2013).

These findings align with sociolinguistic evidence that code-switches are communicatively meaningful (Gumperz, 1982) and commonly used by bilinguals in successful interactions (e.g., Gardner-Chloros, 2009). Together, they challenge the traditional view of code-switching as purely disruptive, instead suggesting that switches may enhance attention, prediction, and memory (see also Valdés Kroff & Dussias, 2023).

Building on this work, we examine whether code-switching benefits extend to recall, a critical memory system requiring active retrieval of information. A previous study found that code-switches heightened bilingual listeners’ attention to information, leading to better recognition memory for that information in a multiple-choice format (Salig et al., 2025). However, it remains unclear whether code-switches similarly boost memory across different contexts and types of learning assessments. Using a free recall paradigm, we test whether bilinguals recall information presented near a code-switch better than information in single-language contexts after listening to short vignettes. We hypothesize that code-switches provide localized memory benefits, improving recall for information presented at or near switch points, without necessarily enhancing memory for entire code-switched vignettes.

### Why and when might code-switches affect memory?

The primary aim of this study is to determine whether bilinguals exhibit improved recall for information presented in code-switched contexts. A secondary goal is to explore the underlying cognitive mechanisms by which code-switches might enhance memory. We consider two distinct accounts.

First, the *inference hypothesis* suggests that bilinguals perceive code-switches as meaningful signals that highlight important information (Gumperz, 1982; Myslín & Levy, 2015; Tomić & Valdés Kroff, 2022). This account involves top-down processing, where listeners draw on prior experience and world knowledge to interpret code-switches as communicatively relevant. The switch serves as a cue, thereby prompting increased attention and deeper encoding of nearby content (Salig et al., 2025).

Second, the *saliency hypothesis* proposes that code-switches enhance memory due to their bottom-up perceptual salience—that is, the noticeable and unexpected phonological and phonetic shifts associated with a code-switch automatically grab attention, leading to enhanced memory encoding.

These two hypotheses propose two qualitatively different cognitive mechanisms by which code-switches may affect bilinguals’ attention and memory. However, both predict that individuals’ code-switching experience will modulate the effect of code-switches on memory—albeit in different ways—providing a basis for distinguishing between them. The inference hypothesis predicts that bilinguals with more code-switching experience will show a greater memory boost from code-switches, as they are better able to infer their communicative relevance. In contrast, the saliency hypothesis predicts the opposite: that bilinguals with less code-switching experience will show a stronger memory effect, as switches are more likely to stand out as perceptually novel and attention-grabbing in this group.

We further test between these two hypotheses by manipulating participants’ beliefs about the motivation behind code-switches. According to the inference hypothesis, bilinguals benefit from code-switches because they assume code-switches are meaningful, for example, emphasizing key information. Thus, the memory benefit of code-switches may rely on listeners believing that switches were produced naturally and therefore meaningfully. If listeners are told that code-switches were artificially inserted (e.g., at the experimenter’s instruction), this may disrupt the inferential process and reduce the memory advantage. Alternatively, if the inference process is automatic and robust to such belief manipulations, listeners may still benefit. By contrast, the saliency hypothesis predicts that memory effects should persist regardless of participants’ beliefs, since they depend on bottom-up, stimulus-driven factors rather than interpretation.

Finally, to explore how real-world experience shapes these effects, we varied the context in which code-switches appeared, comparing memory outcomes for conversational versus narrative scenarios. Under the inference hypothesis, bilinguals may draw on their experiences with code-switching to interpret switches differently across contexts. For example, in conversational scenarios, code-switching often serves pragmatic or social functions, while in narrative contexts, switches may act as stylistic or discursive devices. These distinct functions and contexts could potentially alter the nature of the memory advantage.

### Current study

Bilinguals listened to short vignettes that were either entirely in one language or included code-switches and then verbally recalled each vignette. Our primary prediction was that they would better remember details that were code-switched compared with those presented in a single language. To address our secondary question about the cognitive mechanism behind this effect, we further predicted that the impact of code-switches on recall would interact with bilinguals’ code-switching experience and with their (manipulated) beliefs about the reasons for the switches.

To preview our findings, bilinguals indeed recalled code-switched details better than single-language details. There was also some evidence that code-switching experience modulates this effect, though beliefs about why speakers code-switched did not appear to influence recall.

## Method

This study’s design and analysis plan were preregistered on OSF (https://osf.io/37rqj). All study materials, including links to vignette audio files, data, and analysis scripts are accessible on OSF (https://osf.io/a8fkm).

### Participants

This study took place over two sessions, conducted remotely on participants’ personal devices. Our preregistered target sample size of 180 participants was based on a power analysis, which indicated 95% power to detect the predicted main effect of language context and between 79 and 99% power to detect an interaction between language context and code-switching experience, depending on the interaction’s effect size.

To achieve this target, we screened 382 Spanish–English bilinguals who completed a first-session questionnaire to assess eligibility for the second, experimental session. Participants were recruited through Prolific (https://www.prolific.com/) and paid $15/hour or recruited through the University’s participant pool and received course credit.

The screening questionnaire included the English and Spanish LexTALE vocabulary assessments (Izura et al., 2014; Lemhöfer & Broersma, 2012), the Bilingual Code-Switching Profile (BCSP; Olson, 2024), and a brief language history questionnaire. Bilinguals were eligible for the second session if they (1) identified Spanish and/or English as their first and preferred language, (2) scored at least 55% on both LexTALE assessments, and (3) answered a minimum of 16 out of 20 BCSP questions.

Based on these criteria, 267 bilinguals were invited to participate in the experimental session, and 186 returned to complete it. Seven participants were excluded due to recording issues, and two early participants were excluded because the stimuli were finalized only after their participation. Ultimately, data from 177 bilinguals was analyzed, close to the target of 180.

No participants required exclusion based on preregistered criteria: failing two or more of the three engagement checks (described below), not attempting the story retelling task, or showing no variation in recall accuracy across all vignette details (i.e., scoring 0% or 100% on detail recall accuracy).

The final sample (117 women, 55 men, and five participants of another gender) included bilinguals with diverse language backgrounds. Most participants were likely to be somewhat English dominant, on average having generally learned English at an earlier age than Spanish, scoring high on the English LexTALE assessment, and preferring English (*n* = 118). However, there was considerable variability in our sample: Sixty-two participants learned English before Spanish, 59 learned Spanish before English, and 56 were exposed to both languages from birth. Participants resided in 32 different US states or territories, with the most represented being California (*n* = 44), Florida (*n* = 29), and Texas (*n* = 21)—states with a high degree of Spanish–English bilingualism and code-switching.

Participants reported relatively balanced daily exposure to both languages and moderate code-switching experience (see Table 1). Code-switching experience, as measured by BCSP scores, ranged from 9.38 to 73.96 on a 0–100 scale, with a higher value indicating greater experience with code-switching. Seven participants reported never code-switching, while those who did began doing so at an average age of 7.46 years (*SD* = 5.57).

**Table 1:** Participant characteristics (*N* = 177)

	Mean (*SD*)
Age (years)	30.82 (10.85)
AoA English (years)	1.96 (3.52)
AoA Spanish (years)	4.23 (7.13)
English LexTALE score (out of 100)	92.06 (8.16)
Spanish LexTALE score (out of 100)	67.96 (9.88)
Language exposure entropy (from 0 to 1.58)	0.84 (0.28)
BCSP score (out of 100)	50.37 (13.87)

AoA indicates age of acquisition. LexTALE tests assess written vocabulary and morphological knowledge; English vs. Spanish LexTALE scores are not directly comparable, and LexTALE assessments may underestimate verbal proficiency. Language exposure entropy was calculated based on participants’ self-reports of what percentage of their daily time on average they are exposed to English, Spanish, or other languages (higher indicates more balanced exposure; Gullifer & Titone, 2020). BCSP indicates Bilingual Code-Switching Profile, which measures code-switching experience (higher indicates more code-switching experience)

### Materials

#### Vignettes

Five vignettes were used in this study. Three vignettes were narratives adapted from *Alice in Wonderland* vignettes used in Fraundorf and Watson (2011). For each narrative vignette—Cave, Duchess, and House—we used the same 14 critical details identified by Fraundorf and Watson to code recall accuracy. Originally, all three narratives focused on Alice, but for this study, only the Cave vignette retained its original characters. The Duchess and House vignettes were modified to replace character names and details, removing connections to *Alice in Wonderland* to minimize interference from recurring characters.

Two conversational vignettes—the Airport and Cruise vignettes—were adapted from actual bilingual conversations in the Bangor–Miami corpus (conversations sastre4 and zeledon14; Deuchar et al., 2014). These vignettes featured two bilingual speakers discussing specific topics. Edits were made to ensure that each vignette had self-contained context and included 14 specific details chosen as critical items, which were relatively evenly distributed throughout the conversation. These 14 critical details were used to code recall accuracy, consistent with the narrative vignettes adapted from Fraundorf and Watson (2011).

Each narrative vignette had three versions: one entirely in English, one entirely in Spanish (translated from the original English), and one with code-switches from English to Spanish at seven of the 14 critical details. Each conversational vignette had two versions: one entirely in English and one with code-switches from English to Spanish at seven critical details.

For Spanish versions of the narrative vignettes and English versions of the conversational vignettes (where portions were originally in Spanish), the first author translated the texts. Two native Spanish speakers (who also recorded the vignettes) then reviewed the translations to ensure natural language flow.

#### Code-switches

The code-switches consisted of intrasentential switches where noun or verb phrases switched from English into Spanish. The two bilingual speakers provided feedback on code-switch naturalness. After our initial review, a norming study with 20 Spanish–English bilinguals (age: *M* = 19.25 years, *SD* = 1.55) was conducted to assess the naturalness of our 35 experimental code-switches across the five vignettes and 34 intentionally ill-formed code-switches. Participants were recruited from the University’s participant pool and rated naturalness on a 9-point scale. They were proficient in both languages (Spanish LexTALE *M* = 58.96, English LexTALE *M* = 80.94), had moderate code-switching experience (BCSP *M* = 53.16), and high daily language exposure entropy (*M* = 0.81). The experimental code-switches were rated significantly higher in naturalness (*M* = 6.92, *SD* = 2.04) than the ill-formed code-switches (*M* = 5.03, *SD* = 2.68, *p* < 0.01, η_p_^2^ = 0.61). There was a trend suggesting participants with more code-switching experience rated experimental code-switches as more natural (*r* = 0.41, *p* = 0.07). Minor edits were made to improve poorly rated code-switches. The final version of all vignettes, including code-switched versions, are available on OSF (https://osf.io/a8fkm).

#### Vignette audio recordings

Two Spanish–English bilingual women (ages 19 and 20) with parents from Argentinian backgrounds recorded the vignettes. Both speakers were born in and currently resided in the United States. One was a simultaneous bilingual who frequently code-switches, while the other learned English from age 3 and only occasionally code-switches with bilingual family and friends.

Recordings were conducted in a quiet room using a RØDE cardioid condenser microphone and an Avid Mbox. The same speaker recorded all language conditions for each vignette. Specifically, one speaker recorded all versions of the Cave and House vignettes, while the other recorded all versions of the Duchess vignette. Both speakers participated in the Airport and Cruise conversational vignettes, with each speaker playing the same role across all language conditions.

Vignettes ranged from 90 to 158 seconds long, with conversational vignettes generally longer than the narrative vignettes. Code-switches were produced naturally; audio editing was used only to remove errors and extraneous pauses.

### Design and procedure

Participants completed the study remotely, with the experimental task conducted on PCIbex (Zehr & Schwarz, 2018) and a post-experiment questionnaire on Qualtrics. In PCIbex, participants provided consent, completed brief tasks to verify headphone use (Woods et al., 2017) and microphone functionality, read instructions, and engaged in the story retelling task. The instructions included a four-sentence sample vignette, illustrating the level of detail desired in their retellings, and requested that participants refrain from taking notes while listening to the vignettes. Participants were informed that they could retell the vignettes in English and/or Spanish.

Participants listened to five vignettes, each containing 14 key details. Each participant heard two vignettes in English only, one in Spanish only, and two that included code-switching, in that sequence. We opted for a fixed order of condition to avoid introducing the belief manipulation (described below) before it was necessary. Vignettes were balanced across conditions, so each vignette was presented approximately equally often in each of its language conditions across participants. Participants were assigned to one of 48 lists, which balanced vignette order and the belief manipulation group. The main comparison of interest was between recall of code-switched vignettes/details versus single-language vignettes/details. Additionally, single-language vignettes in both Spanish and English allowed us to determine if baseline recall differed by language, informing interpretation of any code-switching effects.

There was only one code-switched version of each vignette: Seven details were in code-switched sentences, and seven details were in English-only sentences. Although the specific details that appeared in code-switched versus single-language form were not counterbalanced within each vignette, subsequent analyses showed that these details were equally memorable when presented in single-language contexts (see the “Single-Language Memorability of Details” subsection under Results).

Not every sentence in each vignette contained one of the 14 critical details; some sentences did not include a critical detail. This design choice allowed us to include an equal number of code-switched and single-language details in the code-switched versions of vignettes, while maintaining a natural overall proportion of code-switching.

Before hearing each vignette, participants were informed that the speaker was summarizing a book they had read (for narrative vignettes) or that two friends were having a conversation (for conversational vignettes). After listening to each vignette, participants pressed a button to begin retelling it and had up to 75 seconds to complete their retelling. A warning appeared on screen after 70 seconds to indicate that time was almost up. The time limit was necessary to manage audio file sizes and ensure successful uploads to the data server.

#### Belief manipulation

After hearing three single-language vignettes, participants read a belief manipulation text before listening to two code-switched vignettes. Ninety-two participants were assigned to the “Natural belief” group and 85 to the “Instructed belief” group. Those in the Natural belief group were told that the bilingual speakers would switch languages as they naturally would when talking to friends, indicating that speakers had autonomy in their code-switching choices.

In contrast, participants in the Instructed belief group were told that speakers switched languages when prompted by a red circle appearing on a screen, at intervals designed to be random. They viewed an example video showing this prompt and were informed that: “The speakers told us that it was a bit unnatural or odd to have to switch languages when they saw the red circle and that they wouldn’t necessarily have chosen to switch at those points themselves. As you hear the vignettes, try to focus on the content and don’t worry too much about the language switches.” This manipulation was intended to lead the Instructed belief group to believe that code-switches were randomly cued and that speakers had no agency in determining which content was code-switched. Notably, listeners were told that once the prompt appeared, speakers had to switch languages within the sentence, rather than abruptly at a fixed point; this explained why the code-switches sounded grammatical and natural despite supposedly being prompted externally and randomly. Participants in both the Natural and Instructed belief groups listened to identical story audio files.

#### Post-experiment questionnaire

Following the experimental task, participants completed a questionnaire on Qualtrics, which asked participants about prior exposure to the vignettes, how natural the code-switches sounded, familiarity with the speakers’ dialect, their perceptions of why the speakers code-switched, and beliefs about code-switching in general. A key purpose of the questionnaire was to assess the effectiveness of the belief manipulation (e.g., participants in the Instructed belief group should believe that the speakers switched languages as instructed, rather than by choice).

#### Engagement checks

Three engagement checks were embedded throughout the study to confirm participant attention to the task. Two checks, placed in the instructions and the post-experiment questionnaire, asked participants to select a specific response from a set of options. The third, embedded within the story retelling task, asked participants to audio record themselves saying a particular sentence.

At the end of the study, participants were debriefed on the study’s purpose, including the minor deception involved in the belief manipulation.

### Coding

Accuracy in recalling vignette details was scored by two Spanish–English bilingual coders, who were the same individuals who recorded the vignette audio files. Coders were unaware of the language condition assigned to each vignette. Accuracy in recalling each detail was coded as either 0 (not recalled) or 1 (gist recalled). Following the approach of Fraundorf and Watson (2011), we employed a gist coding policy to evaluate memory for key information without requiring perfect recall. Under this policy, details were considered accurately recalled if participants conveyed the main idea, even if they did not recall every element. For example, in one vignette, the coded detail was: “The White Rabbit runs by and drops his fan and gloves.” If participants recalled that the rabbit dropped his things, this was scored as accurate gist recall. If participants only recalled that someone dropped something, this was scored as incorrect gist recall.

To check interrater reliability, approximately 10% of the data (from 18 out of the 177 participants) was coded by both coders. Any questions that arose during coding were discussed among the two coders and the first author to reach a consensus. The two coders achieved 91.78% agreement, with a Cohen’s kappa of 0.84, indicating strong reliability.

## Results

### The effect of code-switches on recall

**Fig. 1:**
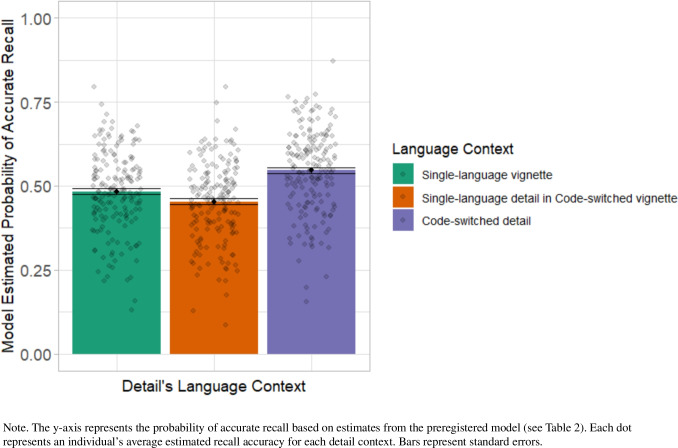
Bilinguals’ recall accuracy by language context of detail. (Color figure online)

As shown in Fig. 1, bilinguals better recalled details they heard in a code-switched sentence (model-estimated probability of accurate recall: 0.55) compared with details they heard in a single-language context—whether in a single-language vignette (0.48) or in a single-language sentence within a code-switched vignette (0.45).

Our preregistered logistic regression (Table 2) confirmed a local effect of code-switching: Within code-switched vignettes, recall was significantly better for code-switched details than single-language details (*p* = 0.02). This effect seemed limited to a local context, as there was no significant overall difference in recall accuracy between single-language vignettes and code-switched vignettes (*p* = 0.22). This finding aligns with our prediction that bilinguals would show improved recall for information presented in a code-switched context.^1^

**Table 2:** Preregistered logistic regression model predicting bilinguals’ recall

Factor	β	*SE*	*z*	*p*
Intercept	− 0.04	0.16	− 0.23	0.82
Global language context: Single-language vs. code-switched vignettes	0.10	0.08	1.22	0.22
Local language context: Single-language vs. code-switched detail (within code-switched vignettes)	0.36	0.15	2.38	0.02
BCSP score	0.00	0.00	− 0.94	0.35
Belief group	− 0.05	0.12	− 0.44	0.66
Global Language Context × BCSP Score	− 0.01	0.00	− 1.90	0.06
Global Language Context × Belief Group	− 0.13	0.10	− 1.31	0.19
Local Language Context × BCSP Score	0.01	0.01	1.50	0.13
Local Language Context × Belief Group	− 0.13	0.14	− 0.94	0.35

Model specification: GistRecallAccuracy ~ LanguageContext × (BeliefGroup + BCSP) + (LanguageContext || ID) + (LanguageContext × (BeliefGroup + BCSP) || Detail). Language context was orthogonally coded, allowing for global and local comparisons

#### Single-language recall by language

Another preregistered analysis assessed participants’ “baseline” recall accuracy for details from single-language vignettes, depending on whether they heard the vignette entirely in English or entirely in Spanish. This analysis included only the single-language narrative vignettes, allowing for a direct comparison (since participants did not hear a Spanish-only version of conversational vignettes).

Participants had higher recall accuracy for details from English-only vignettes (model-estimated probability of accurate recall: 0.57) than from Spanish-only vignettes (0.42), a difference confirmed by the logistic regression (β = − 0.89, *se* = 0.16, *z* = − 5.63, *p* < 0.01). Exploratory analyses suggest that this pattern is related to the sample’s overall English dominance—as recall for English-only vignettes was correlated with English LexTALE scores (*r* = 0.31, *p* < 0.01), and recall for Spanish-only vignettes was correlated with Spanish LexTALE scores (*r* = 0.25, *p* < 0.01). Regardless, the finding that single-language recall was better for English indicates that the memory benefit from English-to-Spanish code-switches is not simply due to better recall for information presented in Spanish; rather, it reflects *an effect specifically tied to the presence of a code-switch*.

#### Single-language memorability of details

Within code-switched vignettes, participants recalled code-switched details more accurately than single-language details (see Fig. 1). To confirm that this effect reflects the presence of code-switches, rather than differences in the inherent memorability of specific details, we conducted an exploratory analysis of baseline memorability using the single-language vignettes. Specifically, we compared recall for details that would appear in code-switched versus single-language form in the code-switched version of each vignette. In the single-language vignettes, recall was nearly identical for both types of details: 0.48 for details that would be code-switched and 0.49 for details that would remain in a single language. A logistic regression model (with random intercepts for participant) predicting gist accuracy in single-language vignettes from this item-level condition (code-switched or not, in the code-switched versions) found no difference (β = 0.03, *z* = 0.67, *p* = 0.50). These findings suggest that the memory advantage for code-switched details is unlikely to be driven by differences in the memorability of specific items, supporting the validity of the design despite the absence of full counterbalancing.

#### Global effect of code-switches on single-language detail recall

Figure 1 shows slightly lower recall for single-language details in code-switched vignettes compared with those in entirely single-language vignettes. To explore whether the global presence of code-switches impairs memory for single-language information, we conducted an exploratory analysis comparing recall for single-language details across these two language conditions. To ensure comparable items, we included only those details that were single-language in *both* the single-language and code-switched versions of the vignettes, excluding any that were code-switched in one version. A logistic regression predicted gist accuracy from global context (code-switched vs. single-language vignette), with intercepts by participant and detail number. This analysis revealed no significant difference in recall (β = 0.05, *z* = 0.84, *p* = 0.40), suggesting that code-switches do not produce a general detriment to memory for surrounding single-language information. However, because the study was not designed or counterbalanced to test this question directly, future work is needed to draw firm conclusions.

### The role of code-switching experience

The main logistic regression (Table 2) found no main effect of bilinguals’ code-switching experience, as measured by BCSP scores, on recall accuracy (*p* = 0.35), nor any interaction with language context (*p* values ≥ 0.06).

**Fig. 2:**
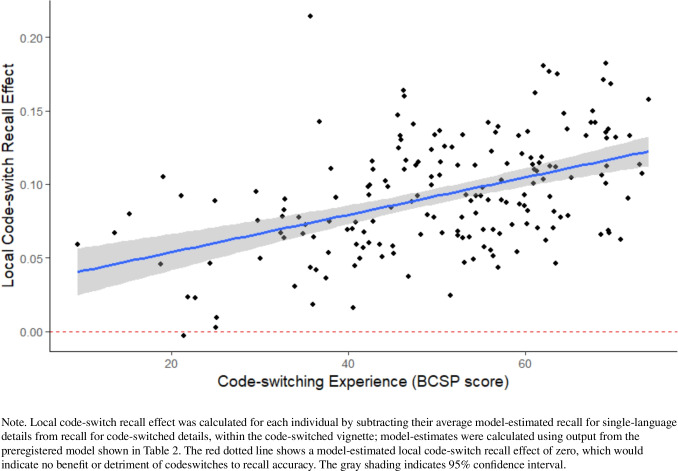
Exploratory correlation: Local code-switch recall effect and code-switching experience. (Color figure online)

Given that the observed interaction effect size (β = 0.01) was smaller than the minimum effect size anticipated in our power analyses (which allowed for a beta as small as 0.03), we conducted an exploratory analysis to assess evidence for a smaller-than-expected interaction between code-switching experience and language context. This simplified analysis allowed us to focus directly on the relationship between code-switching experience and the local memory boost from code-switches, without the additional predictors and random effects included in the main model. For each participant, we calculated a local code-switch recall effect by subtracting their model-estimated recall for single-language details within code-switched vignettes from their model-estimated recall for code-switched details. Positive values indicated a local recall benefit from code-switches. We then correlated local code-switch recall effect with BCSP score. As shown in Fig. 2, individuals with more code-switching experience showed a greater recall boost from code-switches (*r* = 0.46, *p* < 0.01), indicating a significant relationship in the direction predicted by the inference hypothesis. Note, however, that this analysis was exploratory (not preregistered). Regardless, we found no evidence supporting the saliency hypothesis, which predicts that more code-switching experience should lead to *smaller* recall benefits.

### The role of manipulated beliefs about speakers’ code-switches

Approximately half of the participants were informed that the code-switches were produced naturally by the speakers (Natural group), while the other half were told that the speakers were instructed to code-switch at specific points based on a computer program (Instructed group). A post-experiment question showed that those in the Instructed group were significantly more likely to believe that the speakers were instructed to code-switch (*p* < 0.01) than those in the Natural group, suggesting the belief manipulation was effective.

However, the belief manipulation did not affect recall accuracy (Table 2, *p* values ≥ 0.19). Following our preregistration, we reran the main model, substituting belief group with a variable indicating whether participants endorsed the idea that speakers in the study were instructed to code-switch. This approach resulted in highly uneven groups and did not reveal any interactions between belief and language context on recall, so we do not report these results, although they are available on OSF.

In addition to being asked about their beliefs regarding why the speakers *in this study* code-switched, participants were also asked about their beliefs regarding why bilinguals code-switch *in daily life*, selecting multiple options from a list of possible answers. There were no preregistered analyses for this data, but we describe the patterns here. Nearly all of the bilinguals (95%) believed that code-switching is used in daily life when the speaker forgets a word. Critically, the majority of participants also endorsed believing that speakers code-switch for pragmatic or meaningful reasons, such as to highlight information (69%), signal their identity (43%), or as a storytelling device (68%)—suggesting that bilinguals consciously believe that code-switches can be purposeful and serve communicative functions.

### The role of vignette type

Our design included narrative and conversational vignettes. Although we did not have specific predictions regarding how code-switches might affect recall differently across these contexts, we preregistered an analysis to explore potential differences by vignette type. This model included each detail’s language context, vignette type, their interaction, and random effects.

**Fig. 3:**
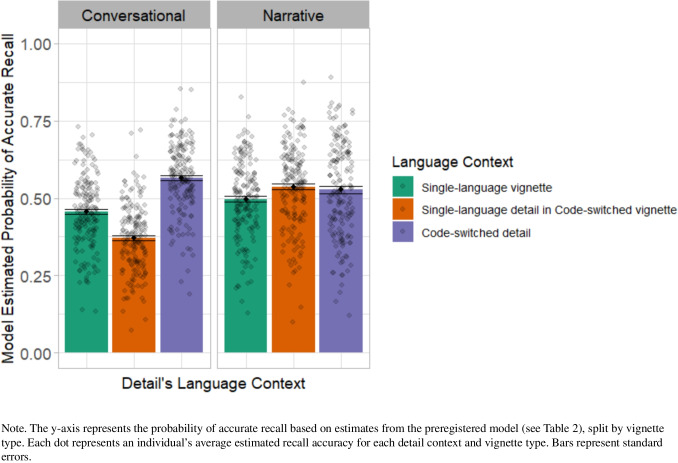
Bilinguals’ recall accuracy by language context of detail and vignette type. (Color figure online)

As shown in Fig. 3, bilinguals exhibited a large local effect of code-switches in conversational vignettes, but showed almost no such effect in narrative vignettes. Despite this apparent pattern, a preregistered model including vignette type as a predictor revealed only a local effect of code-switches, consistent with the main model (Table 2), and did not reveal any effect of vignette type or interaction between vignette type and language context (*p* values ≥ 0.36). This null result may be due to limited power for this analysis or characteristics of our stimuli. Nonetheless, the numeric pattern—though not statistically significant—suggests that context type may be a useful dimension to consider in future studies of bilingual language processing.

## Discussion

As predicted, bilinguals demonstrated improved recall for information presented in naturalistic speech when it was embedded in code-switched sentences, compared with single-language sentences. Prior work (Salig et al., 2025) suggests that code-switches may trigger attentional shifts in bilinguals, orienting them to the speech immediately surrounding the switch and enhancing memory encoding for that information. Here, we show that this attention reorientation and memory benefit occur across varied contexts (e.g., in shorter conversational vignettes, not only in long narrative stories) and across different memory assessment types (i.e., in free recall, not just multiple-choice). This distinction is crucial because active recall, unlike recognition tasks, has consistently been shown to facilitate long-term retention (e.g., the testing effect; Roediger & Karpicke, 2006), suggesting that code-switches might actively enhance learning when memory retrieval is required, as in educational contexts.

Our results extend findings in the literature on code-switching, reinforcing the view that code-switches can be beneficial cues that support comprehension and challenging the perception of code-switches as purely costly. Our study complements other work (Blair & Morini, 2023; Read et al., 2021; Salig et al., 2025; Tomić & Valdés Kroff, 2022; Valdés Kroff & Dussias, 2023) indicating that code-switches benefit predictive processing, attention, and memory encoding in bilingual comprehenders. While code-switches are often associated with short-term processing costs, our findings suggest that they may also yield longer-term comprehension benefits, such as enhanced recall. This raises the possibility that what appears to be a “cost” at the millisecond level may, in fact, reflect deeper processing or attentional engagement that benefits comprehension over time. In essence, focusing solely on the moment-by-moment processing of bilingual code-switching neglects an important adaptive behavior that stems from bilingual modes of interactive communication. This approach brings lab findings into greater alignment with sociolinguistic and pragmatic work on code-switching and provides a crucial link in understanding the apparent disconnect between lab-based switch costs with the ubiquitous presence of bilingual code-switching. To further strengthen this link, future research could investigate whether traditional “switch costs” may actually reflect the attentional reorientation needed to derive these long-term memory benefits. Additionally, exploring the effect of code-switching on memory over extended periods, such as weeks or months, could illuminate its potential benefits in classroom settings.

Indeed, this research suggests that code-switches could have practical applications for learning in educational contexts. Since code-switches naturally tend to coincide with complex or significant information (Calvillo et al., 2020; Myslín & Levy, 2015), they may guide listeners’ attention to important content. While additional research is required to determine how these effects generalize to more naturalistic learning environments, our findings suggest that allowing bilinguals to code-switch in educational settings could facilitate comprehension and memory retention. Future work should explore whether code-switches can improve learning in bilingual classrooms, small-group study sessions, and second-language contexts, where code-switching might support both attention to and recall of crucial information. Of course, the goal of such work should not be to prescribe when or how teachers should code-switch to optimize learning. Instead, it should highlight the benefits of welcoming diverse language practices into classrooms rather than enforcing strict English-only policies.

### Recall benefit is specific to code-switches

Our findings highlight that the memory benefits were specific to code-switched content rather than extending to entire code-switched vignettes. This localized effect suggests that the recall advantage stems from cognitive shifts that occur specifically around code-switches. Crucially, this benefit cannot be explained by any general language preference. Although participants recalled single-language content in English better than in Spanish, they recalled English-to-Spanish code-switched sentences even better than English-only ones. This pattern suggests that the memory boost is driven by the presence of a code-switch itself, rather than by the language in which the information is presented.

One limitation of this study is that it included only English-to-Spanish code-switches. Although we posit that the observed effects are not dependent on switch direction, it is possible that our participants—who seemed, on average, English-dominant—allocated more attention/effort to processing their less dominant language in the code-switched context, resulting in a direction-specific effect. Although we did not obtain a direct measure of language dominance, we explored this possibility by examining whether the code-switch recall effect varied systematically across bilingual profiles that approximate language experience and, by extension, aspects of dominance. We ran exploratory correlation analyses between individuals’ local code-switch recall effect (recall for code-switched details minus recall for single-language details within code-switched vignettes) and measures of language experience: LexTALE scores and percent daily exposure in each language. These analyses yielded no significant correlations (*|r*| values ≤ 0.13, |*p*| values ≥ 0.09). We also assessed local code-switch recall effects separately by first language group (English first, Spanish first, or both from birth) and observed a similar recall benefit of code-switches across all three groups (plot available on OSF). These exploratory findings suggest that the local code-switch recall effect was broadly present across bilinguals with varied language backgrounds and is unlikely to be driven by language dominance (at least in this population). A similar lack of dominance effects was also reported by Salig et al. (2025) and is consistent with other work showing facilitatory or adaptive effects during the processing of code-switches that are not modulated by language dominance (e.g., Tomić & Valdés Kroff, 2022).

### Considering mechanisms for code-switching’s memory benefit

Our results suggest that bilinguals might direct attention to content near code-switches, enhancing encoding. The inference hypothesis posits that bilinguals learn (perhaps implicitly) from experience that code-switches often emphasize meaningful content, prompting this attentional shift and recall boost. This hypothesis predicts a stronger memory benefit for bilinguals with greater code-switching experience and a greater impact when bilinguals believe speakers choose to code-switch. Alternatively, the saliency hypothesis suggests that code-switches capture attention due to their phonological distinctiveness, predicting stronger effects for those with less code-switching experience and no dependency on beliefs about speakers’ agency over switches.

Our main statistical model did not provide decisive support for either hypothesis, as there was no significant interaction between code-switching experience and language context on memory outcomes. However, an exploratory analysis revealed a significant positive correlation: Bilinguals with greater code-switching experience exhibited a larger memory benefit. Although exploratory, this finding aligns with the inference hypothesis, which predicts that code-switching experience contributes to memory effects. Notably, this pattern contradicts the saliency hypothesis, which predicts a *negative* correlation between code-switching experience and memory benefits.

Importantly, further evidence favors the inference hypothesis over the saliency hypothesis. Specifically, monolingual listeners, who lack code-switching experience, did not show the same attentional increases at code-switches as bilinguals in prior work (Salig et al., 2025). This is notable because if perceptual salience alone were driving these effects, then monolinguals—who can presumably detect phonetic shifts—should have exhibited similar attentional responses. Their lack of such responses suggests that attention and memory benefits are not solely due to stimulus-based mechanisms. Instead, a certain level of bilingual experience—and the ability to infer meaning or speaker intent from code-switches—appears necessary to support enhanced memory.

However, the belief manipulation showed no impact on memory, regardless of whether participants believed speakers had agency over code-switching. Although participants’ responses suggested they accepted the manipulation at a conscious level, attentional responses to code-switches may be grounded in deeper, experience-based inferences that are automatic and resistant to temporary experimental manipulations. This automaticity may explain why our belief manipulation failed to shift memory outcomes: If the inference that code-switching signals important information is automatic, it could operate independently of explicit beliefs. Additionally, questionnaire data revealed that participants widely endorsed the belief that code-switching is meaningful in real-world contexts, suggesting that they knew about the communicative functions of code-switching, which may have overridden the experimental manipulation. These findings highlight the challenge of altering well-established attentional mechanisms.

In sum, our findings are inconsistent with the saliency hypothesis and instead point to the inference hypothesis as a promising account for future research to further investigate.

### Exploring contextual influences

An additional consideration for future research is whether the role of code-switches varies by context. While the memory benefit was numerically larger in conversational vignettes, this effect was not statistically significant. We caution against overinterpreting this trend, especially given the null result and the fact that our study was not specifically designed to detect differences between vignette types. Nonetheless, we encourage future work exploring how situational context may shape cognitive effects of code-switches.

Indeed, it seems likely that bilinguals infer different meanings for code-switches depending on context, which may in turn affect memory outcomes. For instance, code-switches in conversational settings might more readily signal important or emotionally salient information, whereas code-switches in narrative or lecture contexts may be less expected and thus less meaningful. Additionally, switch direction may indicate different meanings based on other sociolinguistic factors such as majority-minority language status or community norms (Blokzijl et al., 2017; Torres Cacoullos & Travis, 2019). These distinctions are worth exploring in future work, as is the distinction between being a passive listener (as in our study) versus an active interlocutor, which may affect memory dynamics (e.g., Brown-Schmidt et al., 2024).

## Conclusion

This study demonstrates that bilinguals have better recall for information heard in code-switched sentences, compared with single-language sentences. Although the mechanism behind this effect requires further clarification, the evidence supports the potential of code-switches to aid memory in bilinguals due to their high informativeness and pragmatic intent. This foundational work points to exciting avenues for understanding and leveraging code-switching in learning contexts, potentially offering practical benefits for bilingual education and comprehension, as well as offering empirical evidence that shifts away from deficit-framed perspectives on bilingual language use.

## Data Availability

The study materials and data are available on OSF: https://osf.io/a8fkm/.

## Footnotes

1 Ninety-four participants reported familiarity with at least one vignette—most often the Cave vignette, which retained its original Alice in Wonderland setting. When the analysis was rerun per our preregistration including only the 83 participants (47% of the full sample) who indicated they had never encountered any of the vignettes before, the pattern of results was similar. However, due to reduced statistical power, no effects or interactions reached significance in this subset.

## Code availability

The R analysis script is available on OSF: https://osf.io/a8fkm/.

## Declarations

### Open practices statement

This experiment was preregistered prior to data collection (https://osf.io/37rqj). Study materials, data, and analysis scripts are available on OSF: https://osf.io/a8fkm.
